# Impact of Low-Level Laser Therapy on Orthodontic Tooth Movement and Various Cytokines in Gingival Crevicular Fluid: A Split-Mouth Randomized Study

**DOI:** 10.7759/cureus.42809

**Published:** 2023-08-01

**Authors:** Prasanth Prathapan Santhakumari, Vighnesh Varma Raja, Jacob Joseph, Anjitha Devaraj, Eunice John, Navin Oommen Thomas

**Affiliations:** 1 Department of Orthodontics and Dentofacial Orthopedics, Indira Gandhi Institute of Dental Sciences, Kothamangalam, IND; 2 Department of Orthodontics and Dentofacial Orthopedics, Pushpagiri College of Dental Sciences, Thiruvalla, IND

**Keywords:** orthodontic tooth movement, low-level laser therapy, interleukin, gingival index, cytokines, canine distalization

## Abstract

Background and objectives: A few investigations have detailed the influence of low-level laser therapy (LLLT) on orthodontic tooth movement (OTM), with varying results. The objectives of this study were twofold: to assess the impact of LLLT on OTM and various cytokine levels in gingival crevicular fluid and to contrast the pain levels experienced by patients receiving orthodontic treatment with and without LLLT.

Materials and methods: This split-mouth randomized controlled prospective study comprised 40 patients with an average age of 19.7±2.4 years with Angle Class I malocclusion combined with bimaxillary protrusion who were advised for extraction of the maxillary first premolar and bilateral canine distalization. The control-side canine was distalized solely by the coil spring. On the test arm, a low-level gallium-aluminum-arsenide semiconductor diode laser operating at 980 nm and 100 mW with a continuous-wave energy of 8 J/cm^2^ was used. The canine distalization on either side was measured with a digital caliper following the first premolar extraction (TO), one month after treatment (TOTM1), two months later (TOTM2), and three months later (TOTM3). The gingival index and the level of various cytokines were determined by an enzyme-linked immunosorbent assay at the beginning of the study, on the third and seventh days, and at four, eight, and 12 weeks following the canine distalization. The intra-group and inter-group comparisons were carried out using one-way analysis of variance (ANOVA) and t-tests, respectively, at a 5% significance level.

Results: The results show a highly statistically significant difference in the extent of canine distalization in the test group (TOTM1=2.92±0.44; TOTM2=1.04±0.1; TOTM3​=0.62±0.21 mm) in contrast to the control group (TOTM1=3.23±0.8; TOTM2=2.65±0.2; TOTM3​​​​=2.11±0.24 mm) (p<0.01). After three months of canine distalization, the laser and control group had 34 and 27 patients with mild gingivitis, respectively. Interleukin-1β and interleukin-6 concentrations surged with values of 0.74±0.13 and 0.049±0.001 pg/g at seven days following treatment in the laser group, respectively. The difference in tumor necrosis factor concentration between the groups was shown to be highly statistically significant in all treatment phases (p<0.001). The differences in the epidermal growth factor and microglobulin levels were found to be statistically significant within both groups from T0 to T5. The average visual analog scale (VAS) scores at several subsequent evaluations of the laser and control groups were found to be highly statistically significant.

Conclusion: The findings imply that when the periodontal microenvironment is stimulated by orthodontic force, several paramount cytokines are released, triggering an ordered sequence of biological processes that appear to expedite OTM with reduced associated pain.

## Introduction

The initial phase of orthodontic tooth movement (OTM) is characterized by a brief inflammatory reaction marked by vasodilation and an influx of leukocytes from the periodontal microvasculature [1,2]. The process of bone resorption can also be attributed to the production of inflammatory agents, such as prostaglandin E and interleukin (IL)-1β, both of which collaborate with bone cell membranes. Cytokines are low-molecular-weight peptides that are produced in autocrine and paracrine settings in reaction to localized stimuli, such as stress exposure, and have an impact on the typical physiologic processes of bone turnover and remodeling [3]. Professionals may find it beneficial to keep track of the concentrations of these biochemical indicators following OTM to assess the extent of bone remodeling. Gingival crevicular fluid (GCF) exhibits immunological responses and interactions in response to metabolic stress. ILs are specifically crucial for OTM because they increase osteoclast production and bone resorption. IL-1, IL-2, IL-6, and IL-8 are proinflammatory ILs, while IL-1 receptor antagonist (IL-1RA), IL-4, IL-10, and IL-13 are antiinflammatory ILs. IL-1 is a cytokine that promotes bone resorption, and its level rises a day after the initial onset of OTM [4-6].

Studies of molecular alterations in GCF are vital instruments in OTM because they indicate periodontal health and remodeling. Various cytokines, including tumor growth factor beta 1 (TGF-ß1) and epidermal growth factor (EGF), are synthesized by fibroblasts and stromal cells and have been implicated in the formation of osteoclasts. Furthermore, despite being formed in organs apart from bones, β2-microglobulin (2-MG) increases the affinity of insulin growth factor-I for improving bone production [1]. Tumor necrosis factor alpha (TNF-α) is a common inflammatory agent that has been associated with bone resorption and has been demonstrated to be increased in the context of orthodontic stress [7,8].

OTM is made up of an array of inflammatory-like responses. Any measure that alters these processes has the potential to alter the pace of tooth movement. Many researchers have attempted to establish an approach to expediting tooth movement without traumatizing the periodontium by changing the molecular response to biomechanical pressures. Although the use of chemicals, such as osteocalcin, the active component of 1alpha,25-dihydroxyvitamin D3 (1alpha,25(OH)2D3), and prostaglandin E2, in facilitating OTM has been documented, these chemicals have a systemic impact on the metabolism, inducing localized discomfort after alveolar bone injections, limiting their usage in OTM [9]. Furthermore, orthodontic therapy is a time-consuming technique that typically lasts 20 to 30 months. As a result, patient compliance declines, and a range of adverse effects, such as bone resorption and caries, occur. To reduce the length of orthodontic therapy, the rate of tooth movement must be increased [7].

Low-level laser therapy (LLLT) is advantageous and effective in lowering orthodontic discomfort by blocking the release of pain receptors and accelerating OTM. The biological stimulation of tissues in the mouth triggered by LLLT additionally occurs through the intracellular assimilation of the laser beam by the tissue of interest, which results in the amplification of intracellular signaling sequences, enhancing the metabolic process and having anti-inflammatory effects on the oral tissues [10]. A few investigations detail the influence of LLLT on OTM. However, the findings vary due to differing laser characteristics, dosages, locations, and durations of laser administration. To achieve the best outcomes, more research into accurate and specialized radiation exposures is required [11].

The objectives of this study were twofold: to assess the impact of LLLT on OTM and various cytokine levels in GCF and to contrast the pain levels experienced while receiving orthodontic therapy with and without LLLT.

## Materials and methods

Patients presenting to the Department of Orthodontics and Dentofacial Orthopedics of Pushpagiri Institute of Medical Sciences, Tiruvalla, India, were enrolled in this split-mouth randomized controlled prospective study. The study was performed after obtaining approval from the Pushpagiri Institute of Medical Sciences Ethics Committee with Institutional Review Board number IRB/2/15/2022 and following the Helsinki Declaration, and informed consent was collected from the study subjects [12]. Forty patients (21 males and 19 females) between 18 and 25 years with an average age of 19.7±2.4 years were investigated for the effects of LLLT on the rate of OTM, the concentrations of cytokines in GCF, and the levels of pain reported.

All patients presented with Angle Class I malocclusion combined with bimaxillary protrusion and were advised for extraction of the maxillary first premolar and bilateral maxillary canine distalization. Every participant had good oral hygiene, a probe depth of no more than 3 mm, mild gingivitis based on the gingival index, no radiologic indications of alveolar bone loss, and no previous use of anti-inflammatory medicines in the preceding month of the research. Before commencing the study, oral hygiene instructions were delivered. Patients with systemic disorders or medications that may interfere with bone metabolism were excluded from the trial.

Following a preliminary screening of 81 patients, 41 were eliminated on account of failure to comply with the inclusion criteria (n=28) or refusal to take part in the study (n=13). Employing G*Power statistical software (Heinrich Heine University Düsseldorf, Germany), the sample size was computed with an effect size of 0.50, i.e., α=0.05, and a statistical power of 0.80 for the amount of canine distalization in millimeters. The minimal sample size determined for the analysis was 34 teeth per group. To account for potential dropouts, the study included 40 elements per group.

All patients were allocated using a computerized random number generator and a basic randomization procedure. Each area was concealed in a series of sealed and labeled envelopes and assigned either of two treatments (test or control). The random allocation process and intervention assignments were carried out by a single clinician who had no involvement in the future phases of the experiment. A total of 80 maxillary canines were chosen and analyzed for the study (40 on each side).

All patients received fixed orthodontic treatment with stainless steel (SS) brackets ranging in size from 0.022 to 0.028 inches (Ormco Corp., California). Following the initial alignment and leveling stage, a final SS wire of 0.016x0.022 was put in place, and the first premolar extractions were carried out. Then, after seven days, a segmented arch of 0.016 to 0.022 ss was used in conjunction with a closed coiled nickel-titanium spring (length of 9 mm, G&H Orthodontics, USA) that provided a force of 50 N at its greatest extension. The buccal aspect of the spring was used to close the space between the first premolars. A commercially available dynamometer was used to gauge the force generated by the spring. Then, the spring was gradually reactivated every month until the extraction space was completely closed.

The control-side canine was distalized solely by the coil spring. Meanwhile, on the test arm, the LLLT was performed with a low-level gallium-aluminum-arsenide semiconductor diode laser functioning at 980 nm (Wiser Laser Doctor Smile, Italy) and 100 mW with a continuous-wave energy of 8 J/cm^2^. With the laser point in contact with the gingiva, the beam of the laser was delivered at a total of 10 points, for 10 seconds at each point, at subsequent periods: onset of canine retraction = three days, seven days, four weeks, eight weeks, and 12 weeks thereafter. Sites for laser application included the distobuccal gingival crest, the mesiobuccal gingival crest, a central spot on the labial surface concerning each of the other sites, and two sites at the apex of the tooth parallel to points 1 and 2, respectively. The same spots were chosen for use on the palatal surface.

**Figure 1:**
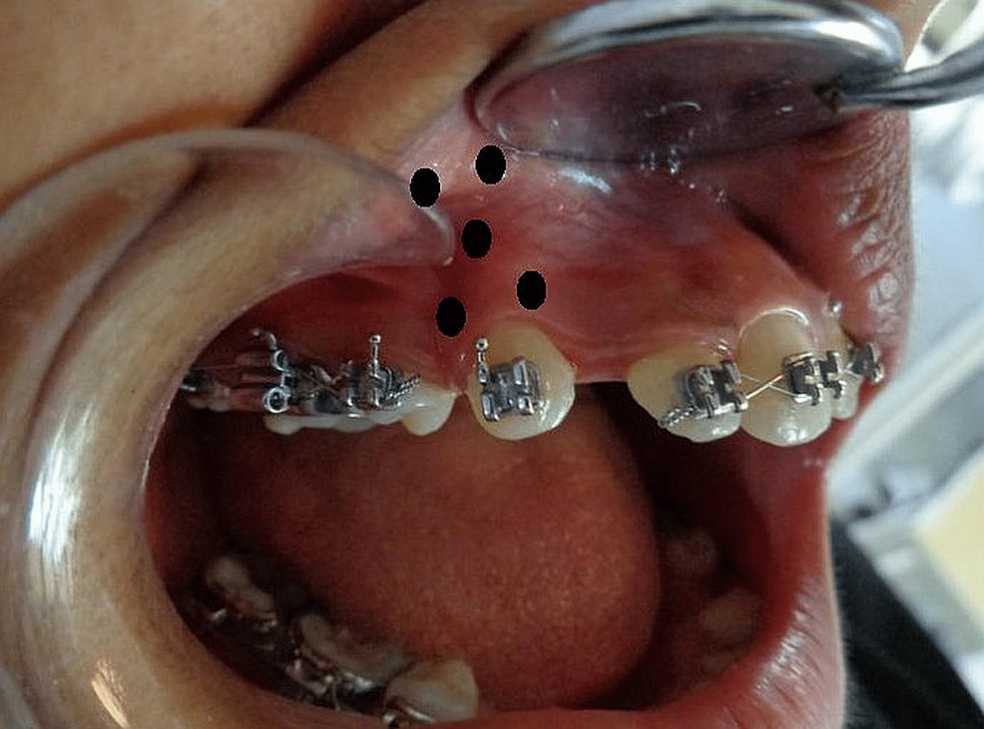
Markings for the low-level laser therapy

Following irradiation at the aforementioned periods, a single examiner measured the tooth displacement on either side with a digital caliper (Mitutoyo, Kawasaki, Japan) following the first premolar extraction (T0), one month shortly after treatment initiation (TOTM1), two months (TOTM2), and three months (TOTM3). Similar to previous investigations [9,13], the extent of canine distalization in millimeters was measured by employing a digital caliper to determine the distance between the point of the canine cusp and the mesiobuccal cusp tip of the first molar and documented as distance (Figure 1).

**Figure 2:**
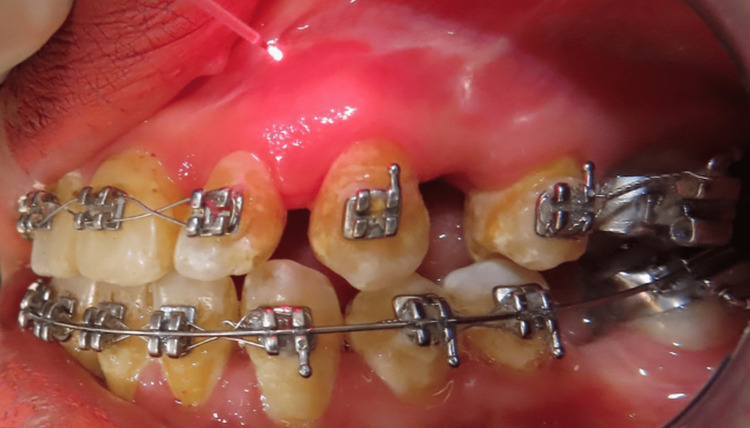
Low-level laser therapy

**Figure 3:**
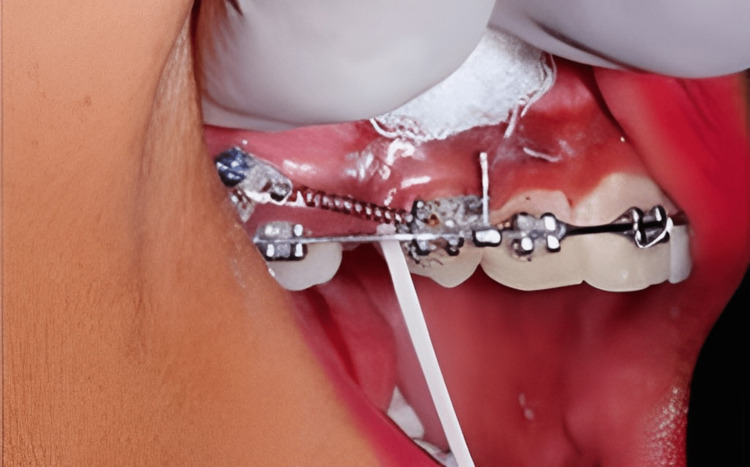
GCF collection for analysis GCF: gingival crevicular fluid

Distalization was performed on the control side without the use of LLLT. A sheath encasing the laser probe had a transparent plastic tip that was replaced on the control arm for false irradiation with a sheath with a black plastic tip. The operator utilized laser safety glasses to prevent the laser sheath from being identified. The use of the laser and the placebo sheath tubing was overseen by a third investigator. Gingival index results were documented before collecting the GCF. The GCF sample was carried out using the Offenbacher et al. [14] approach. GCF was taken from both sides in a similar manner (Figures 2, 3).

The tooth was carefully rinsed with water, and the research sites were sealed off with cotton pellets and air-dried. Paper strips (Harco, Tustin, CA) were gently inserted 1 mm into the gingival sulcus and kept in place for 30 seconds. After one minute, another strip was inserted at the same location. The mechanical injury was avoided by taking precautions. A Periotron (Harco, Tustin, CA) was used to determine the quantity of GCF in the periopaper. The paper strips from each location were kept at -30°C until they were finally processed. To extract the sample thoroughly from the paper strips, we centrifuged the GCF from the periopaper using aliquots of a solution (50 mM buffered phosphate at pH 7.2, comprising inhibitors of protease, 0.1 mM phenylmethylsulfonyl fluoride, and 50 g/mL leupeptin, pepstatin, and antipain each). Henceforth, 100 L of the previously mentioned buffer was added to the strip, and the tube was centrifuged at 15,000 g for five minutes. The centrifugation was then performed again with an additional 100 L.

The GCF collected from the two strips yielded a volume measuring 200 µL, which was then kept at -30°C for further analysis. The protein level of the extract was calculated using the Bradford technique with bovine serum albumin as a reference [15]. GCF was extracted at the following periods on both canines: T0, before beginning canine retraction; T1 and T2, upon three and seven days of canine retraction in conjunction with and without LLLT on either side, respectively; T3, T4, and T5 after four, eight, and 12 weeks of canine retraction alongside and without LLLT on both sides, respectively. IL-1β and IL-6 levels in the samples were determined using a two-site sandwich enzyme-linked immunosorbent assay (ELISA). A two-site ELISA was used to determine the EGF. TNF and β2-MG were measured using a sandwich enzyme immunoassay (EIA) using solid-phase polystyrene-beaded immobilized antibodies tagged with 3-D'Galactosidase, as described by Mogi et al. [16]. All the specimens were tested twice.

Primary and secondary outcome assessment

The primary outcome was the total time required for the distalization of the maxillary canines, expressed in millimeters. This distance was measured on the study cast by a single examiner four times: at baseline, following the first premolar extraction (TB), one month after treatment initiation (TOTM1), two months (TOTM2), and three months (TOTM3) later. An alginate impression was used for making study casts at four time points (TB, TOTM1, TOTM2, and TOTM3).

The pain experienced following OTM was the secondary outcome. At the beginning of the study, each patient was prompted to rate their discomfort on a visual analog scale (VAS). Following comprehensive guidance, the patients were instructed to fill out their VAS ratings at baseline, the third and seventh days following treatment, and one month, two months, and three months thereafter. A score of 0 indicated that there was no pain or discomfort, but a rating of 10 indicated that there was excruciating discomfort. Every patient was recalled six months after intervention by the same examiner for clinical and radiographic assessments. To examine the reliability, at TOTM1, 10 study subjects were selected at random, and measurements were duplicated. For each follow-up session, the kappa coefficient was calculated, and an acceptable level of reliability (intraclass correlation coefficient (ICC)=0.86) was documented for each assessment.

Statistical analysis

IBM SPSS Statistics for Windows, Version 26 (Released 2019; IBM Corp., Armonk, New York, United States) was utilized for data analysis. The statistical significance was established at p≤0.05. One-way analysis of variance (ANOVA) was applied to compare cytokine values at each recorded time interval within each group. A paired t-test was utilized for the comparison of cytokine concentration levels, pain intensity VAS scores, and canine movement between groups.

## Results

The study consisted of 40 individuals, one of whom failed to follow up at the T3 phase and three at the T4 phase. As a result, the final evaluation consisted of 36 participants. The mean±standard deviation (SD) of the distalization distance in both groups at various phases are shown in Table 1. The student's t-test was employed to determine the statistically significant difference in distance between the groups through TOTM1 to TOTM3. The results show that the laser group experienced more canine distalization than the control group at all three treatment phases (TOTM1, TOTM2, and TOTM3). After one month, the mean measure of distalization of the canines in the test and control arms was 2.92±0.44 mm and 3.23±0.8 mm, respectively, with a statistically significant difference (p=0.04). During the second and third months of therapy, a statistically highly significant difference in the extent of canine distalization in the laser group (TOTM2=1.04±0.1 mm; TOTM3=0.62±0.21 mm) in contrast to the control group (TOTM2=2.65±0.2 mm; TOTM3=2.11±0.24 mm) (p<0.01) was also found (Table 1).

**Table 1:** Mean± SD of canine retraction between the study groups at different time intervals TB: baseline following the first premolar extraction; TOTM1: one month after treatment initiation; TOTM2: two months after treatment initiation; TOTM3: three months after treatment initiation; SD: standard deviation

Time point	Test group (mm)	Control group (mm)	t-test	p-value
T_B_ (n=40)	3.87±0.81	3.85±0.74	0.11	0.90
T_OTM1 _(n=39)	2.92±0.44	3.23±0.8	-2.12	0.04
T_OTM2 _(n=36)	1.04±0.1	2.65±0.2	-43.2	<0.001
T_OTM3 _(n=36)	0.62±0.21	2.11±0.24	-28.03	<0.001

Except for T1 (three days following the commencement of treatment), the gingival index revealed a statistically significant difference (p<0.05) in the extent of gingivitis between groups. At the culmination of three months of canine distalization, the laser group had 34 patients with mild gingivitis, while the control group had only 27 subjects with mild gingivitis (Table 2).

**Table 2:** Frequency distribution of the severity of gingivitis based on the gingival index between the study groups at different time intervals T0: before beginning canine retraction; T1 and T2: three and seven days of canine retraction, respectively; T3, T4, and T5:  after four, eight, and 12 weeks of canine retraction, respectively

Time points	Degree of gingivitis	Test group n (%)	Control group n (%)	Total n (%)	Chi-square test	p-value
T_1 _(n=40)	Mild	32	30	62	0.29	0.87
	Moderate	4	5	9		
	Severe	4	5	9		
	Total	40	40	80		
T_2 _(n=40)	Mild	36	26	62	8.04	0.02
	Moderate	2	11	13		
	Severe	2	3	5		
	Total	40	40	80		
T_3 _(n=39)	Mild	36	26	62	8.18	0.02
	Moderate	2	11	13		
	Severe	1	2	3		
	Total	39	39	78		
T_4 _(n=36)	Mild	34	26	60	6.84	0.03
	Moderate	1	8	9		
	Severe	1	2	3		
	Total	36	36	72		
T_5 _(n=36)	Mild	34	27	61	6.25	0.04
	Moderate	1	8	9		
	Severe	1	1	2		
	Total	36	36	72		

IL-1β concentrations surged with the highest mean (0.74±0.13 pg/g) levels at T2 in the laser group and gradually decreased later. Meanwhile, the control group had the highest mean (0.60±0.3 pg/g) IL-1β levels at T2 and returned to normal levels over the T4 and T5 (Table 3).

**Table 3:** Varying levels of cytokines in GCF during OTM between and within the study groups T0: before beginning canine retraction; T1 and T2: three and seven days of canine retraction, respectively; T3, T4, and T5:  after four, eight, and 12 weeks of canine retraction, respectively; GCF: gingival crevicular fluids; OTM: orthodontic tooth movement; IL-1β: interleukin one beta; TNF-α: tumor necrosis factor α; EGF: epidermal growth factor; β2-MG: β2 microglobulin; ANOVA: analysis of variance

Variables	Time point	Test group	Control group	t-test	p-value
IL-1β	T_0 _(n=40)	0.58±0.12	0.58±0.27	0	1
	T_1 _(n=40)	0.69±0.15	0.59±0.25	2.17	0.03
	T_2 _(n=40)	0.74±0.13	0.60±0.3	2.71	0.008
	T_3 _(n=39)	0.62±0.32	0.60±0.14	0.36	0.72
	T_4 _(n=36)	0.60±0.3	0.58±0.26	0.30	0.76
	T_5 _(n=36)	0.61±0.25	0.59±0.15	0.41	0.68
	p-value (ANOVA)	0.013	0.998		
IL-6	T_0 _(n=40)	0.039±0.002	0.039±0.002	0	1
	T_1 _(n=40)	0.048±0.007	0.041±0.004	0.5.05	<0.001
	T_2 _(n=40)	0.049±0.001	0.040±0.003	17.07	<0.001
	T_3 _(n=39)	0.041±0.003	0.04±0.002	1.70	0.09
	T_4 _(n=36)	0.04±0.007	0.039±0.003	0.79	0.43
	T_5 _(n=36)	0.04±0.008	0.04±0.007	0	1
	p-value (ANOVA)	<0.001	0.191		
TNF-α	T_0 _(n=40)	0.47±0.11	0.45±0.2	0.55	0.58
	T_1 _(n=40)	1.74±0.25	0.50±0.24	22.63	<0.001
	T_2 _(n=40)	1.25±0.34	0.50±0.14	12.9	<0.001
	T_3 _(n=39)	0.96±0.21	0.46±0.23	10.02	<0.001
	T_4 _(n=36)	0.83±0.13	0.44±0.17	10.93	<0.001
	T_5 _(n=36)	0.61±0.09	0.43±0.19	5.13	0.001
	p-value (ANOVA)	0.001	0.49		
EGF	T_0 _(n=40)	0.14±0.1	0.18±0.1	1.79	0.08
	T_1 _(n=40)	0.68±0.19	0.48±0.13	5.49	<0.001
	T_2 _(n=40)	0.62±0.1	0.52±0.2	2.83	0.006
	T_3 _(n=39)	0.61±0.05	0.57±0.11	2.07	0.04
	T_4 _(n=36)	0.44±0.07	0.40±0.17	1.3	0.2
	T_5 _(n=36)	0.22±0.91	0.20±0.51	0.11	0.91
	p-value (ANOVA)	0.001	0.001		
β2-MG	T_0 _(n=40)	0.38±0.81	0.37±0.97	0.05	0.96
	T_1 _(n=40)	8.42±2.54	3.44±5.01	5.61	0.0001
	T_2 _(n=40)	11.25±2.83	6.02±3.72	7.08	<0.001
	T_3 _(n=39)	17.89±3.7	6.32±3.11	14.95	<0.001
	T_4 _(n=36)	20.21±3.69	8.28±4.7	11.98	<0.001
	T_5 _(n=36)	21.03±4.55	9.07±2.19	14.21	<0.001
	p-value (ANOVA)	0.001	0.001		

At T1 (p=0.03) and T1 (p=0.008), there were significant differences between the laser and control groups. Through the T3 to T5 treatment phases, there were insignificant differences in IL-1 values between the laser and control groups (p>0.05). Nevertheless, one-way ANOVA demonstrated a statistically highly significant difference (p=0.013) within the laser group from T0 to T5, but an insignificant difference was recorded in the control arm (p=0.998).

The mean IL-6 level for the test side was significantly greater on the third (0.048±0.007 pg/g) and seventh (0.049±0.001 pg/g) days in contrast with baseline (0.039±0.002 pg/g) and were confirmed to be statistically highly significant throughout all treatment stages in the laser group (Table 3). The mean IL-6 concentration within the control arm was determined to be statistically negligible (p>0.05). On the third and seventh days of the treatment phase, there were significant variations in IL-6 levels between the groups (p<0.01).

The mean TNF-α for the test arm was significantly greater (1.74±0.25 pg/g) at T1 compared to TB (0.47±0.11 pg/g), subsequently declining to 0.61±0.09 pg/g at T5. Within the laser group, the difference in TNF-α concentrations was determined to be statistically highly significant (p<0.001). Similarly, the difference in TNF-α concentrations between the groups was shown to be statistically highly significant in all treatment phases except T0 (p<0.001). In the laser group, the mean EGF concentration steadily elevated from 0.14±0.1 pg/g at T0 to 0.22±0.91 pg/g at T5. Meanwhile, the average EGF level in the control group attained a high level at T3 (0.57±0.11 pg/g) from 0.18±0.1 pg/g at T0 and then declined to 0.20±0.51 pg/g at T5. The difference in EGF level was found to be statistically significant within both groups from T0 to T5 (Table 3) and between the groups at T1, T2, and T3 (p<0.001).

The mean β2-MG increased over time to its peak at T5 (21.03±4.55 pg/g in the laser and 9.07±2.19 pg/g in the control groups) from baseline values of 0.38±0.81 pg/g in the test and 0.37±±0.97 pg/g in the control groups. Throughout all treatment periods from T1 to T5, a statistically significant difference was established in MG levels between the experimental and control groups. Furthermore, one-way ANOVA demonstrated a statistically significant difference between the groups from T0 to T5. Our findings show that the rising levels of measured cytokines constitute relatively specific biological changes following OTM.

Table 4 illustrates the average VAS scores at several subsequent evaluations on the laser and control groups.

**Table 4:** Results of the experienced pain levels between the study groups at various time intervals based on the VAS score VAS: visual analog scale

Variables	3 days	7 days	1 month	2 months	3 months
Test group	6±0.46	5.25±0.17	4±0.12	3±0.2	1.5±0.19
Control group	5.8±0.52	6±0.55	5±0.69	4.5±0.27	3±0.24
p-value	0.07	<0.001	<0.001	<0.001	<0.001

During one month of therapy (T3), the laser group exhibited a significant decrease in the mean level of dental discomfort due to distalization (4±0.12) compared to the control group (5±0.69) with a statistically highly significant difference (p<0.001). Furthermore, pain scores were revealed to be considerably lower in the laser group (p<0.001) at T4 (laser group: 3±0.2; control group: 4.5±0.27) and T5 (laser group: 1.5±0.19; control group: 3±0.24).

## Discussion

Our investigation aimed to assess the stimulatory impact of Ga-Al-As semiconductor diode LLLT on OTM, the IL-1β concentrations in GCF on either side, and a decrease in pain levels. Intermittent laser application for 12 weeks significantly increased cytokine levels in the study arm compared to orthodontic force exclusively on the contralateral control arm. This occurred concurrently with increasing rates of OTM, which was consistent with a previous study [17].

Prior human research has shown that the amount of IL-1β in GCF increases considerably during OTM [6,18]. IL-1β has a direct correlation to bone resorption because it causes receptor activator of nuclear factor kappa-B ligand (RANKL) activity in osteoblasts and periodontium and promotes the proliferation of osteoclast precursors. As a result, in this study, we assessed the levels of IL-1β and other cytokines during OTM because they serve as a biologic indicator for assessing the bone remodeling mechanism by triggering bone resorption and also form an integral part of the inflammatory reactions associated with orthodontic care.

The increase in IL-1β concentrations from T0 to T5 on the test arm indicates that LLLT initiated a self-propagating sequence of events. LLLT is directed at mitochondria in the electron transport chain and cellular porphyrins. When light photons get absorbed, they promote adenosine triphosphate synthesis by triggering the electron transport chain and briefly stimulating reactive oxygen species, increasing the process of transformation of adenosine diphosphate to adenosine triphosphate, and subsequently releasing nitric oxide from its binding location. These critical criteria have a substantial impact on the clinical success of LLLT [17].

Prior research has established an elevated gingival index score during fixed mechanotherapy [19]. However, in our investigation, the gingival index score was significantly lowered over the treatment period, notably in the laser group, which could be attributable to LLLT. The extent of canine retraction was considerably higher in the test arm in contrast to the control arm, which was consistent with previous research [17,20]. LLLT offers the added benefit of being less intrusive and less stressful and having a dose-related impact on bone remodeling. It is noteworthy to state that the 8 J/cm^2^ employed in this study for LLLT yielded biostimulatory benefits, including more rapid tooth movement, which was consistent with prior findings [13,17].

LLLT was found to have anti-inflammatory, biostimulatory, and analgesic properties. This can be accounted for largely by more intense stimulation following LLLT, resulting in increased blood flow induced by laser therapy due to improved and normalized homeostasis in cell metabolism. Others have proposed that the anti-inflammatory action is due to the suppression of mast cell degranulation. It is also thought that LLLT stimulates defective cellular activity [21]. It was documented that a laser wavelength of 800 nm, with a power of 0.25 mW and an exposure time of 10 seconds, accelerated OTM 1.3 times faster than the control [22].

Because of its deeper level of penetration and consequent capacity to activate osteoblastic cells on the tissue of interest, a Ga-Al-As diode with a wavelength of 980 nm was employed. Several prior studies employed Ga-Al-As with wavelengths that extend from 650 nm to 860 nm. However, the energy output differed throughout all studies, resulting in inconsistent findings [11]. The conflicting results on GCF variations in several studies may be attributable to variances in the methodology used to gather GCF concentrations [9,23]. While the suction technique utilized for GCF sampling can result in damage and alterations in the absorbed volume, the absorption approach is less invasive and more commonly employed. This approach is carried out using a variety of devices. The standard approach is to use the absorption technique by employing Periopaper as an adsorbent and then measuring the absorbed volume with a Periotron gadget. Periopaper was employed for the absorption of GCF in this study [23].

Age, gender, hormones, pain threshold, and anatomic variances have a large influence on pain and the rate of OTM. As a result, the split-mouth trial was proposed to reduce the possibility of inaccuracy. It does, however, have the drawback of carryover action. To accomplish this, a plastic shield with a wavelength equivalent to the laser was inserted along the midline [11]. It has been stated that the early painful reaction to orthodontic force is caused by the formation of acute inflammation and alterations in the blood circulation in the periodontium. A VAS score was employed to assess pain severity because earlier research has shown it to be accurate and reliable [24].

Assessing the levels of particular inflammatory agents may be a clinically effective noninvasive approach for determining the degree of periodontal remodeling during orthodontic treatment. Furthermore, this knowledge could help the clinician determine the best timing for orthodontic therapy [25]. Because IL-1 is strongly linked with root resorption, it should be explored in larger samples to determine the extent of the phenomenon. In addition, studies on the effects of varying dosages, sustained use of LLLT on OTM, and cellular interactions in the periodontium in association with LLLT are essential. Fernandes et al. also explained that photobiomodulation accelerates tooth movement during molar intrusion, due to the modulation of IL-6, IL-8, and IL-1β during bone remodeling, which is of great help [26]. Chung et al. [27] showed contrary results to previous findings of the effect of laser phototherapy on regulating the rate of OTM as it showed no improvement, which could be a result of less number of patients tested and the time duration of the therapy.

The present study has some limitations, such as the sample size of only 40 patients, which may limit the generalizability of the findings. A larger sample size would increase the statistical power and provide more accurate results. The limitation of a split-mouth trial is that it has carry-across effects, such as contamination or spilling of the effects of one intervention to another. Further studies including a broader panel of cytokines could provide a more comprehensive understanding of the inflammatory response. Longer follow-up periods would be beneficial to assess the stability of the achieved tooth movement and the persistence of pain reduction. Moreover, the study was conducted at a single center, which may limit the external validity of the findings. The replication of the study in different settings and populations would enhance the generalizability of the results.

## Conclusions

The findings imply that when a periodontal microenvironment is stimulated by orthodontic force, several paramount cytokines, such as IL-1β, IL-6, TNF-α, EGF, and β2-MG, are released, triggering an ordered sequence of biological processes. LLT, when combined with orthodontic force, increased GCF secretion of several cytokines, appeared to expedite OTM, and reduced the associated pain with OTM. LLLT at the initial phase of orthodontic therapy has recently become feasible and may have a significant therapeutic advantage in reducing the duration of treatment.
